# Patterns of immunotherapy-induced pneumonitis in patients with non-small-cell lung cancer: a case series

**DOI:** 10.1186/s13256-021-02926-y

**Published:** 2021-07-03

**Authors:** Sarah Picard, Desiree Goh, Ashley Tan, Nisha Sikotra, Eli Gabbay, Tim Clay

**Affiliations:** 1 Sir Charles Gairdner Hospital, Hospital Ave, Nedlands, WA 6009 Australia; 2 St John of God Hospital, 12 Salvado Rd, Subiaco, WA 6008 Australia; 3 Fiona Stanley Hospital, 11 Robin Warren D, Murdoch, WA 6150 Australia; 4 The University of Notre Dame, Fremantle, Australia; 5 St John of God, Subiaco, Australia; 6 Adjunct Associate Professor of Medicine, Edith Cowan University, Joondalup, WA Australia; 7 Bendat Family Respiratory Research and Development Fund, Subiaco, Australia

**Keywords:** Pneumonitis, Immunotherapy, Malignancy, NSCLC, irAE

## Abstract

**Background:**

Immunotherapy has become an efficacious option in the management of solid organ malignancies. Immune-related adverse events including pneumonitis are well described and may be particularly of concern in patients receiving immunotherapy for non-small-cell lung cancer.

**Case presentations:**

In this paper, we describe three cases of immunotherapy-induced pneumonitis occurring in the management of lung malignancy. Our cases include a 54-year-old Caucasian woman with squamous cell lung cancer who was successfully rechallenged with immunotherapy after prior significant pneumonitis, a 65-year-old Caucasian man with metastatic squamous cell lung cancer who developed pneumonitis after multiple cycles of uneventful immunotherapy, and a 73-year-old Caucasian man with squamous cell lung cancer who developed early-onset pneumonitis with rebound on steroid taper.

**Conclusions:**

This case series has provided further insight into the presentation and risk factors for pneumonitis in patients with non-small-cell lung cancer. Each of the cases of immunotherapy-induced pneumonitis illustrates the different potential patterns that may arise when immunotherapy-induced pneumonitis develops. This case series provides key learning points that may assist physicians managing non-small-cell lung cancer with immunotherapy.

## Background

Immunotherapy is increasingly prescribed for solid organ and hematological malignancies, with striking improvements in progression-free and overall survival in some cancers [1]. Immunotherapeutic drugs may cause life-threatening immune-related adverse events (irAEs) including pneumonitis, complicating their use in lung cancer. Patients with advanced non-small-cell lung cancer (NSCLC) have a higher incidence of pneumonitis from immunotherapy (4.7% incidence) compared with the overall incidence of pneumonitis in patients with other solid organ malignancies receiving immunotherapy (2.92% incidence) [2]. In this paper, we describe three cases of immunotherapy-induced pneumonitis occurring in the management of lung malignancy.

Immune checkpoint inhibitors in routine clinical practice today target programmed death 1 (PD-1), programmed death ligand 1 (PD-L1), and cytotoxic T-lymphocyte-associated protein 4 (CTLA-4). These pathways are expressed in normal tissue as a mechanism to control the immune system, and they can become dysregulated in the tumor microenvironment [3]. Inhibition of these proteins with monoclonal antibodies causes upregulation of the immune system [3, 4], allowing it to target and destroy cancer cells [2, 5]. However, T cells may also attack noncancerous cells, resulting in irAEs [5, 6] including pneumonitis.

Pneumonitis is defined as focal or diffuse inflammation of the lung parenchyma [5, 7–9]. Pneumonitis may present asymptomatically and be incidentally found on computerized tomography (CT) scanning, or present symptomatically with cough, dyspnea, fatigue, or chest pain or clinical findings of progressive hypoxemia and respiratory failure [10–12]. Diagnosis is based on appropriate history and suggestive radiological findings on CT scanning. Definitive diagnosis requires a combination of bronchoalveolar lavage and/or a biopsy; however, these investigations are often impractical in an acutely unwell individual [10–12]. Suggestive radiological features include ground glass opacities, interstitial reticulation, or cryptogenic organizing pneumonia-like changes [8-10, 12]. Pneumonitis caused by immunotherapy is graded using the Common Terminology Criteria for Adverse Events (CTCAE) severity scale, which ranges from grade 1, asymptomatic, through to grade 5, where death occurs [2] (Table 1). Initial treatment is often empiric. Treatment options include supportive therapy alone, oral or intravenous corticosteroids, cessation of immunotherapy, and, in refractory cases, the use of steroid-sparing agents as part of prolonged immunosuppression with mycophenolate, infliximab, or cyclophosphamide [11, 13]. The outcome of patients who develop pneumonitis can range from complete resolution to death [12, 13].

**Table 1:** CTCAE grading of pneumonitis with suggested management as per ESMO guidelines

Grade	Clinical features	Management
1	Asymptomatic	Oral steroids—prednisone 1 mg/kg daily or equivalent with taper over 4–6 weeks after recovery Clinical and assessment every 2–3 days initially Delay checkpoint inhibitor until equivalent daily dose of 10 mg oral prednisolone or less
2	Symptomatic—limiting instrumental activities of daily living	As per grade 2 AND Radiological assessment every 2–3 days initially
3	Severe symptoms—limiting self-care activities of daily living	Hospital admission High-dose intravenous corticosteroids (methylprednisolone 2–4 mg/kg/day or equivalent) Cease immunotherapy permanently Commence immunosuppression if no clinical or imaging improvement after 2 days (such as infliximab, mycophenolate mofetil, cyclophosphamide) Wean steroids slowly over 6 or more weeks
4	Life-threatening respiratory compromise	As per grade 3
5	Death	

*CTCAE* Common Terminology Criteria for Adverse Events, *ESMO* European Society for Medical Oncology

## Case presentations

**Fig. 1:**
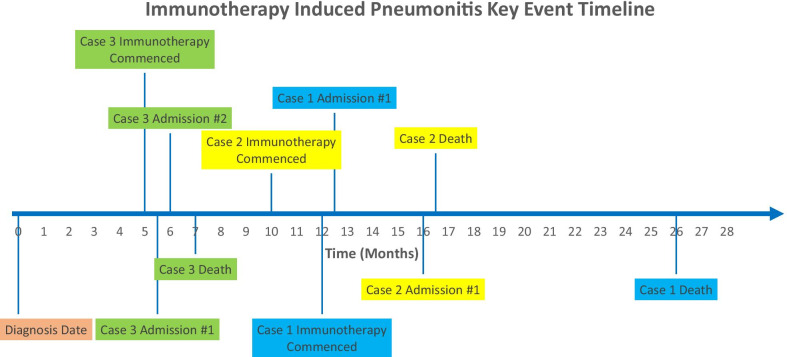
Timeline of key events

We present three separate cases of immunotherapy-associated pneumonitis seen at our institution, each of which we believe has important teaching points, in accordance with the CARE reporting checklist (Fig. 1).

### Case 1: key point—rechallenge with immunotherapy after prior significant pneumonitis

A 54-year-old Caucasian woman with T3N3M0 stage IIIB squamous cell lung cancer was initially treated with induction chemotherapy (carboplatin/paclitaxel) followed by chemoradiotherapy (cisplatin/etoposide) with curative intent. After 6 months, she relapsed in her primary tumor with increased fluorodeoxyglucose (FDG) uptake on her positron emission tomography (PET) scan. She was commenced on the PD-1 inhibitor nivolumab. She had a prior history of localized cervical cancer, hypothyroidism, and a 6 pack-year smoking history. There were no other clear risk factors for pneumonitis. Following her first dose of nivolumab, she immediately experienced facial flushing, fever, myalgia, and night sweats. Nine days following commencement of nivolumab, she was admitted to hospital with dyspnea.

**Fig. 2:**
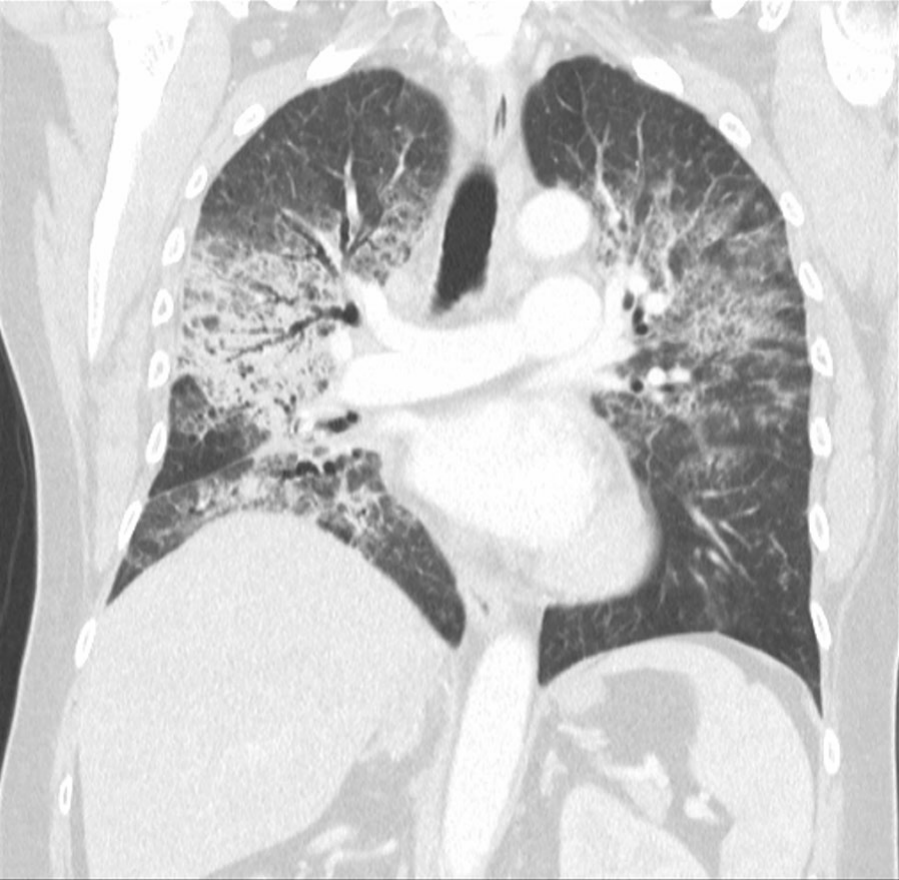
Case 1—chest computed tomography (CT) revealing signs of pneumonitis

On admission, her observations were oxygen saturation 80% on room air, heart rate 116 beats per minute, and respiratory rate 20 breaths per minute. Her CT scan showed extensive patchy ground-glass infiltrates bilaterally (Fig. 2). She received supplemental oxygen via nasal prongs. She was commenced on intravenous methylprednisolone 500 mg daily for 4 days followed by a prolonged taper on oral prednisolone over 3 weeks. She was concurrently treated with intravenous piperacillin–tazobactam and oral trimethoprim/sulfamethoxazole for 6 days to cover for potential concurrent infection. During her admission, other causes of pneumonitis were considered and excluded, and antibiotics ceased. The pneumonitis was grade 3 as per the Common Terminology Criteria for Adverse Events (CTCAE) version 5. Upon discharge, her cardiorespiratory parameters were within normal range, and she had returned to her premorbid function.

**Fig. 3:**
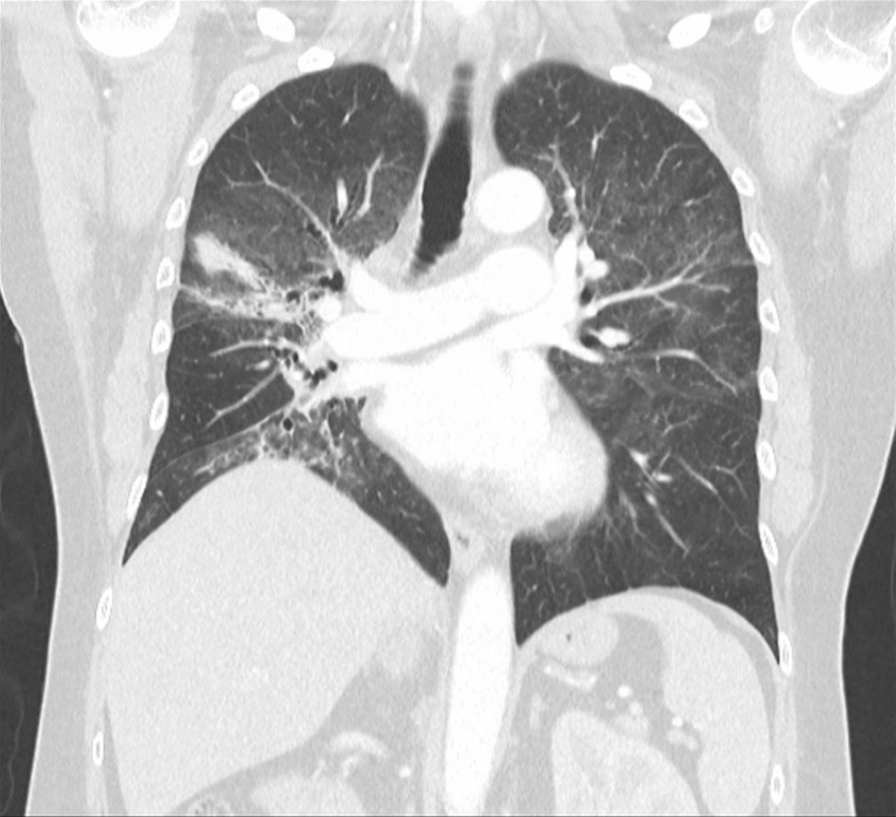
Case 1—CT chest 2 months after commencement of pneumonitis treatment with incomplete resolution of pneumonitis changes

Two months after treatment, the FDG-PET scan showed an initial partial metabolic response, with incomplete resolution of the changes due to pneumonitis (Fig. 3). Over time, the pneumonitis settled, but there was cancer progression on FDG-PET after 5 months off nivolumab. On discussion between the patient and her treating team, nivolumab was reinstituted and treatment continued for a period of 18 months with a best response of stable disease. No further episodes of pneumonitis were observed. The patient subsequently died from cancer-associated venous thromboembolism unrelated to her prior immunotherapy.

### Case 2: key points—pneumonitis occurring after multiple cycles of immunotherapy

A 65-year-old Caucasian man was diagnosed with metastatic squamous cell carcinoma of the lung presenting with a large primary tumor, mediastinal nodal involvement, and a malignant pleural effusion. He was initially treated with carboplatin and gemcitabine chemotherapy with partial response. On progression, he was treated with nivolumab with stable disease as best response. He had a 47 pack-year smoking history and no other significant medical history. The significant smoking history and the subsequent risk of developing pneumonitis were considered, and the decision was made to proceed with immunotherapy and accept the associated risk given the potential benefit treatment would provide. He was commenced on nivolumab and was in his seventh month of treatment at the time of hospital admission. He presented 3 days following administration of nivolumab with dyspnea, productive cough, lethargy, and decreased appetite.

**Fig. 4:**
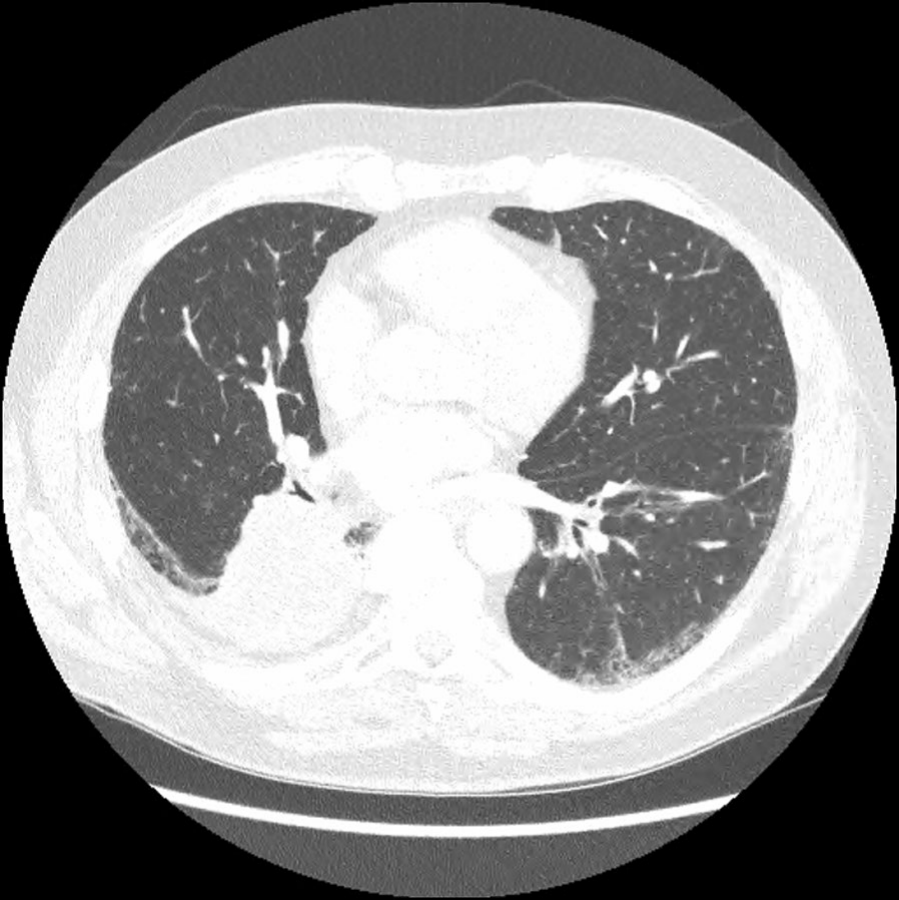
Case 2—CT chest upper lobes revealing signs of pneumonitis, widespread mixed ground glass and interstitial infiltrate. There is progression of malignancy

**Fig. 5:**
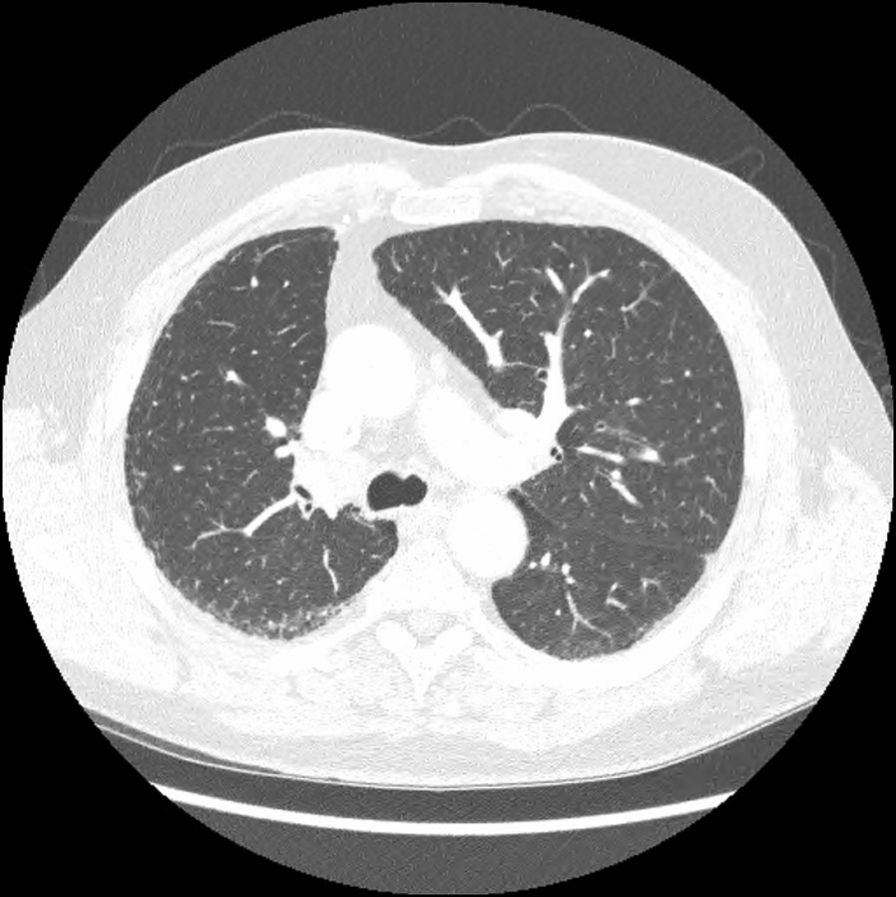
Case 2—CT chest lower lobes revealing signs of pneumonitis, widespread mixed ground glass and interstitial infiltrate. There is progression of malignancy

**Fig. 6:**
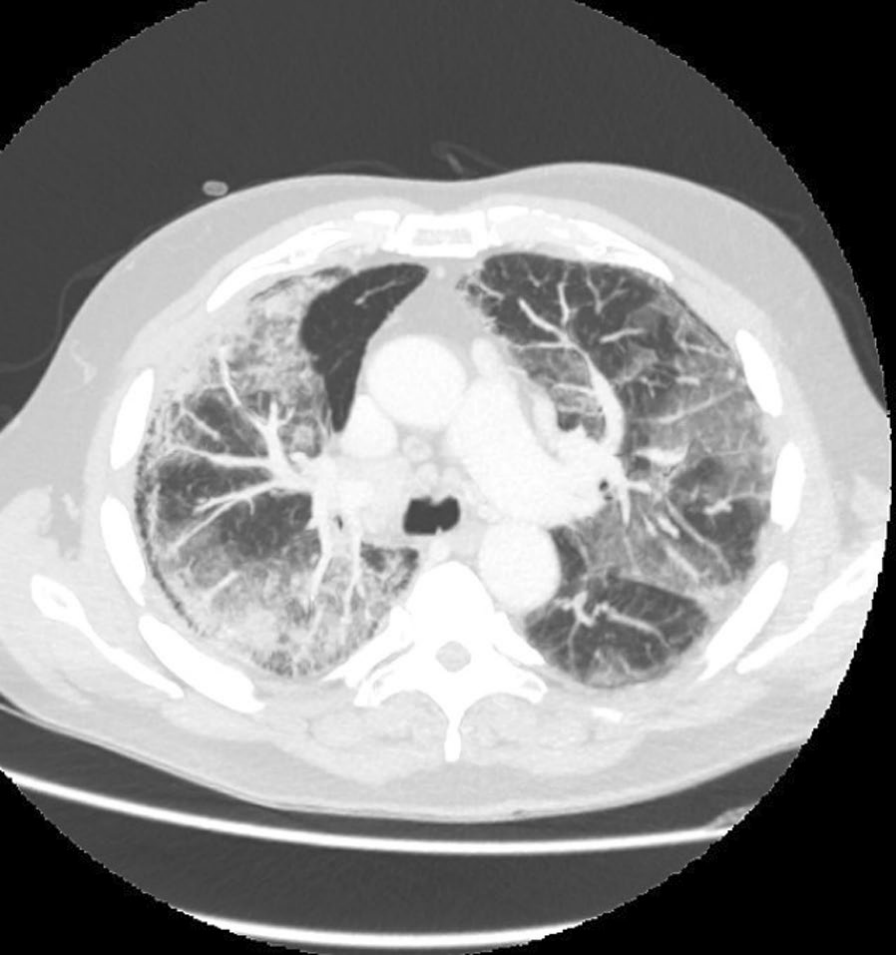
Case 2—CT chest upper lobes with deterioration of pneumonitis despite treatment

**Fig. 7:**
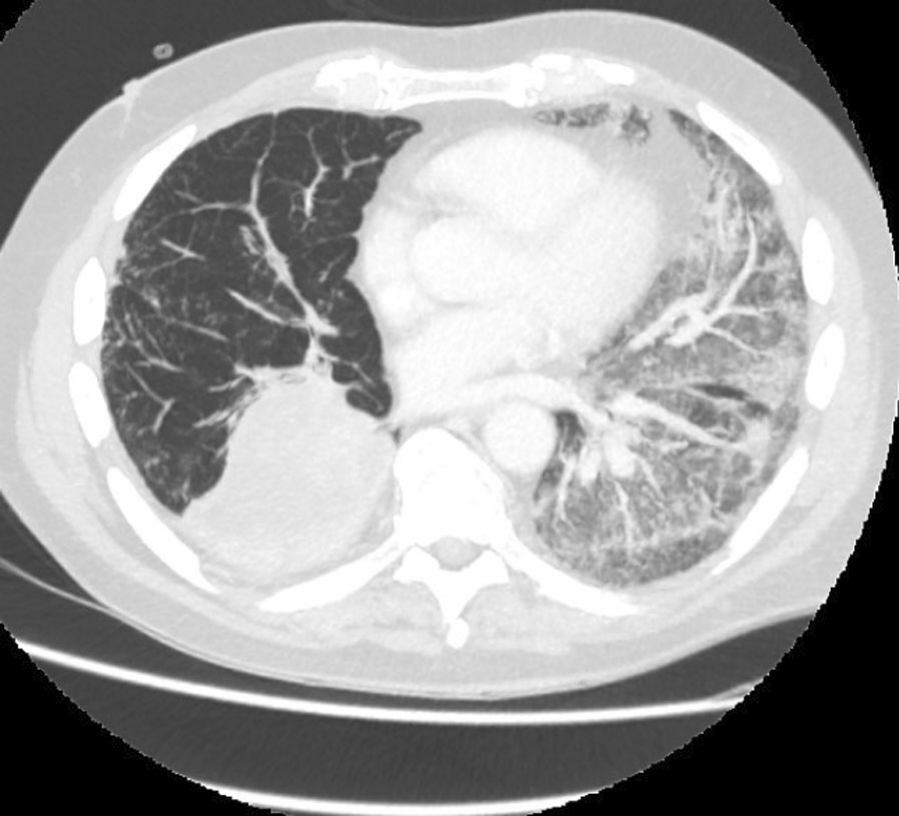
Case 2—CT chest lower lobes with deterioration of pneumonitis despite treatment

On admission, he was hypoxemic with oxygen saturation of 82% on room air, respiratory rate of 21 breaths per minute, heart rate of 80 beats per minutes, and temperature of 38.7 °C. His CT chest showed widespread mixed ground glass and interstitial infiltrate, with progression of his malignancy (Figs. 4, 5). He was treated with intravenous methylprednisolone 1 g for 5 days. Mycophenolate was started on the fifth day of admission for a total of 3 days. He received concurrent intravenous antibiotic therapy with cefepime, meropenem, azithromycin, and trimethoprim/sulfamethoxazole. He continued to deteriorate, with progressively worsening hypoxemia, and was subsequently transitioned to end-of-life care with grade 5 pneumonitis (Figs. 6, 7).

### Case 3: key points—early-onset pneumonitis with rebound on steroid taper

A 73-year-old Caucasian man with was diagnosed with T3N1M0 squamous cell carcinoma of the lung. He received treatment with chemoradiotherapy (weekly carboplatin/paclitaxel) with curative intent. An early FDG-PET scan showed partial response, and consolidation immunotherapy durvalumab, a PD-L1 inhibitor, was commenced. He had a background of emphysema, cerebellar hematoma, cerebrovascular disease, coronary artery disease, hyperlipidemia, and hypothyroidism. He was an ex-smoker with a 75 pack-year history. The significant smoking history, emphysema, and recent radiotherapy were considered as risk factors for the development of pneumonitis. After consideration, treatment proceeded, given the clear survival benefits demonstrated in the PACIFIC clinical trial [14]. Six days following the first dose of durvalumab, he developed dyspnea and a productive cough.

**Fig. 8:**
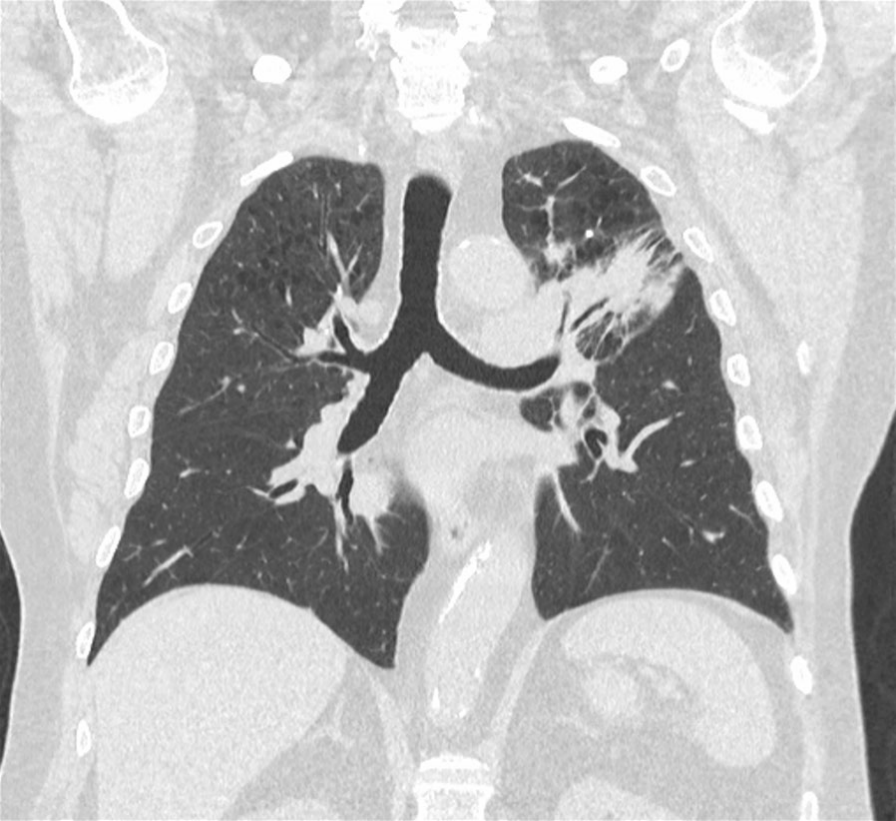
Case 3—CT chest before immunotherapy commenced

**Fig. 9:**
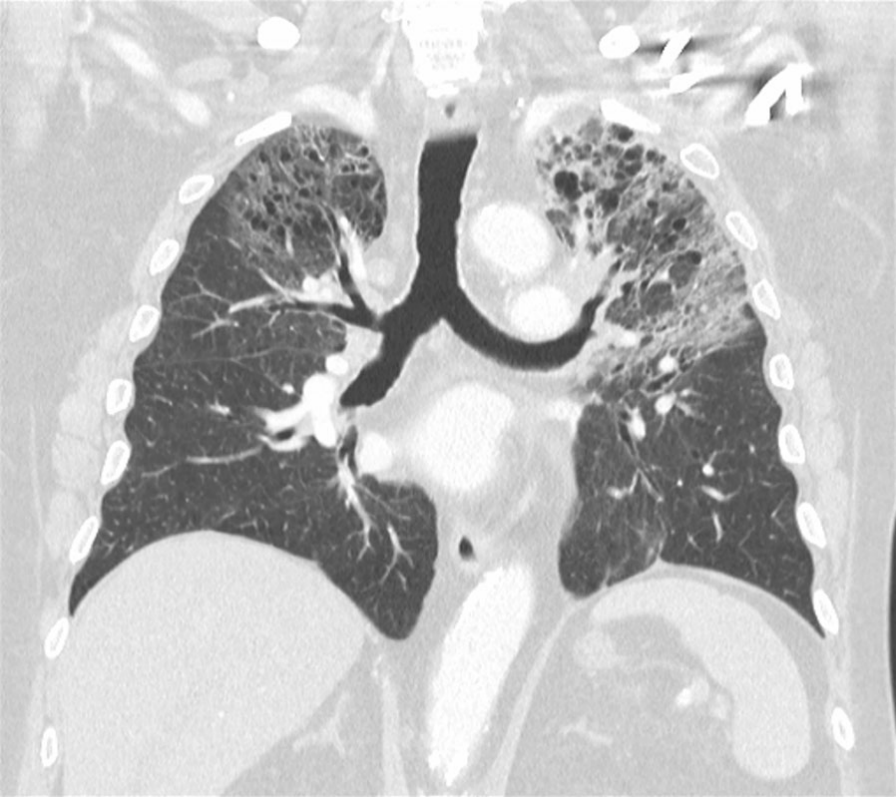
Case 3—CT chest after immunotherapy given revealing signs of pneumonitis, diffuse peribronchial ground-glass changes. There is decrease in size of lung cancer

**Fig. 10:**
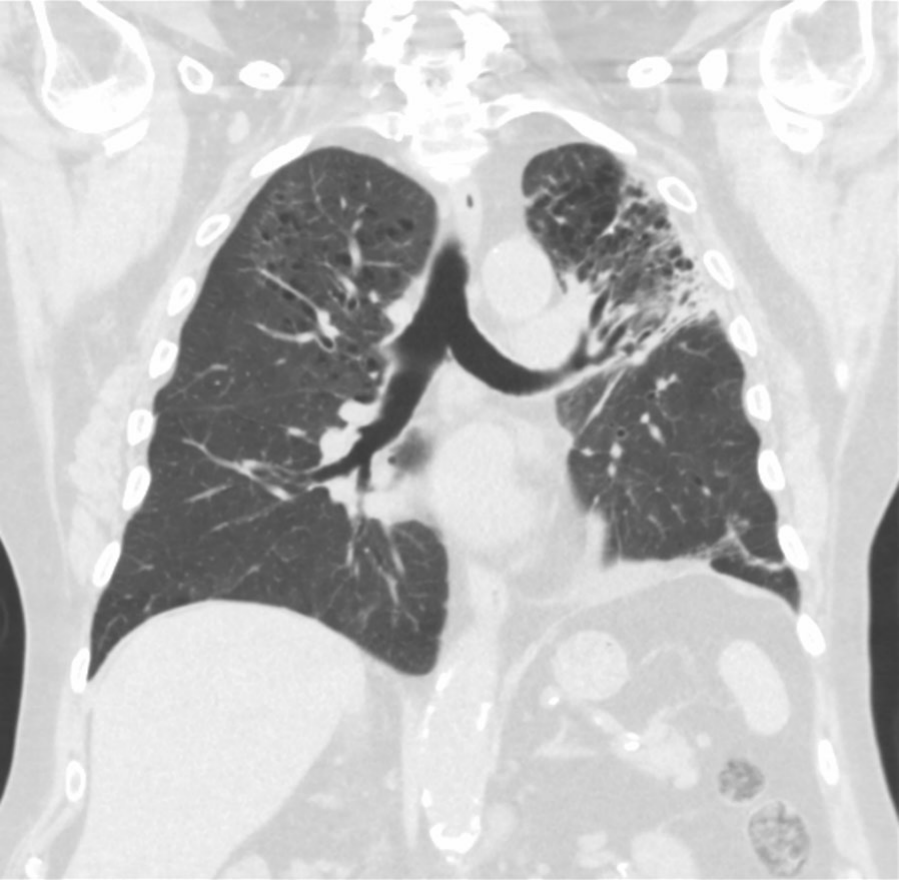
Case 3—CT chest after treatment of pneumonitis with initial recovery

The patient was admitted to hospital. His initial observations showed oxygen saturation was 96% on room air with a respiratory rate of 20 breaths per minute, temperature of 37 °C, and heart rate of 78 breaths per minute. His CT scan of the chest showed diffuse peribronchial ground-glass changes and a decrease in size of the lung cancer (Figs. 8, 9). He was admitted for 6 days and commenced on oral prednisolone 100 mg with a weaning regime and concurrent doxycycline for grade 3 pneumonitis. The patient improved clinically, his observations were within normal cardiorespiratory parameters, and he was discharged on a prolonged steroid taper. The patient was reviewed by his respiratory physician and medical oncologist in clinic 3 weeks after discharge from hospital. Immunotherapy was not recommenced. A repeat CT scan showed marked resolution of the ground-glass changes, and he had returned to his premorbid function (Fig. 10).

**Fig. 11:**
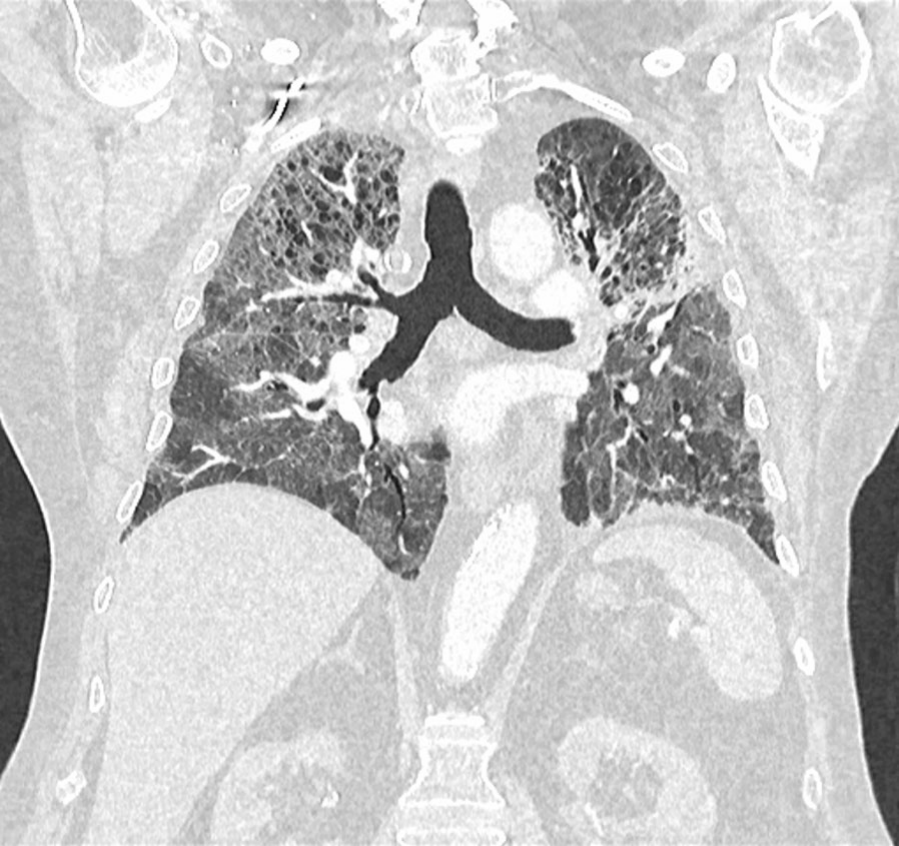
Case 3—CT chest after relapse of pneumonitis. There is no change in size of lung cancer

One week after clinic review, the patient was readmitted to hospital with increasing dyspnea and hemoptysis. He had been on corticosteroids for a period of 4 weeks and 4 days at this time. He was hypoxemic at 92% oxygen saturation on 4 L of oxygen per minute via nasal prongs, had a respiratory rate of 20 breaths per minute and heart rate of 95 beats per minute, and was afebrile. A repeat CT scan of the chest showed recurrence of diffuse ground-glass infiltrates, in keeping with acute pneumonitis, and no change in the size of the lung cancer (Fig. 11).

On readmission, oral corticosteroids were reescalated to prednisolone 100 mg. The patient rapidly deteriorated, with worsening hypoxemia requiring admission to Intensive Care Unit (ICU). Immunosuppression was increased to pulse methylprednisolone 1 g daily with subsequent administration of intravenous cyclophosphamide. Broad antimicrobial therapy was administered to cover for concurrent infection (including ceftriaxone, piperacillin–tazobactam, meropenem, trimethoprim/sulfamethoxazole, and azithromycin over the period of his admission). Respiratory support included supplemental oxygen via high-flow nasal prongs and periodic noninvasive ventilation. Intubation and mechanical ventilation were deemed inappropriate in view of the guarded prognosis and in discussion with the patient and his family. Despite maximal therapy, the patient’s condition deteriorated, and he died from grade 5 pneumonitis 18 days following admission.

## Discussion and conclusion

Checkpoint inhibitor immunotherapy now enjoys widespread use in cancer care. Severe adverse events with single-agent PD-1 or PD-L1 inhibition are uncommon but well described—they can be unpredictable with regard to timing and severity and can result in significant morbidity and, occasionally, mortality. With any presentation, irAEs must be considered in the differential diagnosis and acted on promptly. Pneumonitis remains a toxicity of particular concern for clinicians treating NSCLC with greatest incidence of immunotherapy-induced pneumonitis [2]. In the current context, Coronavirus 19 infection also remains an important differential, and in jurisdictions with community transmission, it is particularly important to exclude this diagnosis urgently.

The first case highlights that immunotherapy rechallenge is possible but must occur after consideration of the therapeutic options and a discussion on risks and benefits. It is important for clinicians to highlight the uncertainty about the risk of further episodes of irAEs on immunotherapy rechallenge.

Our case series demonstrates:

1. Patients with underlying lung pathology or smoking history are susceptible to developing high-grade pneumonitis.
2. Immunotherapy-induced pneumonitis can present at any time after commencement of immunotherapy.
3. The severity of immunotherapy-induced pneumonitis does not appear to be correlated with the timeframe of onset.
4. Immunotherapy-induced pneumonitis can flare or recur during the period of steroid taper.
5. Following careful consideration, immunotherapy rechallenge can occur for some patients with prior immunotherapy-induced pneumonitis. A careful discussion of the potential risks and benefits is required with the patient, given the uncertainty about the risk of further episodes of irAEs on immunity rechallenge.

Nishinu *et al*. identified that pneumonitis more commonly occurs in patients who have NSCLC 4.1% and renal cell carcinoma 4.1%, compared with patients with metastatic melanoma 1.6% [1]. Similarly, a meta-analysis of 16 randomized controlled trials found that the lowest rates of pneumonitis were seen in patients treated for advanced melanoma (0.72%) and highest in patients treated for advanced NSCLC 4.7% [2]. Patients with NSCLC are more likely to have underlying lung disease such as chronic obstructive pulmonary disease (COPD), potentially increasing their susceptibility to pneumonitis and/or to developing higher-grade pneumonitis [12]. A recent study by Suzuki *et al*. [15] indicates that lower lung static volumes such as with large thoracic tumors are associated with higher pneumonitis risk post-immunotherapy [16]. A past or current smoking history may also be a risk factor for the development of severe pneumonitis. Whether this is due to smoking itself or due to smoking-related lung diseases is unclear and perhaps difficult to discern, with two studies showing smoking on its own was not associated with an increased risk or incidence of pneumonitis [17, 18].

Pneumonitis was demonstrated in this case series to present at variable time frames following administration of immunotherapy. The onset of pneumonitis from time of administration of immunotherapy can be variable, which can complicate and delay the diagnosis [19]. In a study by Nishino *et al*., the median time to onset of immunotherapy-induced pneumonitis was 2.6 months, ranging from 0.5 to 11.5 months [13]. Pneumonitis must be considered among the differential diagnosis for patients presenting with early clinical deterioration and respiratory symptoms early in their treatment course. However, late onset of irAEs including pneumonitis can occur more than 90 days after cessation of immunotherapy [20]. Delaunay *et al*. [9] found that the time to onset of pneumonitis was shorter in patients with NSCLC compared with melanoma, with a median time to onset of 2.1 and 5.2 months, respectively. In the same study, they found that the time to onset and severity of pneumonitis appeared to have no correlation [9]. As pneumonitis has a widely variable and unpredictable onset, pneumonitis must be carefully considered in the differential diagnosis of any patient who has received immunotherapy presenting with dyspnea, hypoxia, and/or cough, regardless of the time of onset.

Management of pneumonitis is based on the grade attributed by clinicians using the CTCAE definitions [19]. Treatment options include observation, oral or intravenous corticosteroids, cessation of immunotherapy, and, in refractory cases, the use of immunosuppression drugs including mycophenolate, infliximab, and cyclophosphamide [11, 13]. Patients may require admission to hospital and respiratory support in a high-dependency or intensive care unit. The outcome of patients who develop pneumonitis can range from complete resolution to relapse, palliation, or death and is dependent on a multitude of variables [12, 13]. The mainstay of therapy is corticosteroids; however, steroid-refractory cases challenge clinicians’ decisions regarding the appropriate dose, timing, and route of administration [19]. Steroid-sparing agents are often employed in the case of steroid refractoriness; however, there appears to be limited data on the efficacy of these treatments [11]. The length of treatment and weaning regime implemented can also be a challenge with rebound toxicity on withdrawal of steroids [19]. As per the European Society for Medical Oncology guidelines, prednisolone should be weaned over 6 weeks minimum in grade 2 pneumonitis or minimum 8 weeks in grade 3 or 4 pneumonitis to prevent rebound of pneumonitis symptoms. However, despite a prolonged weaning regime of immunosuppression following an episode of pneumonitis, pneumonitis may relapse without further exposure to immunotherapy, with cases of chronic immune checkpoint pneumonitis as described by Naidoo *et al*. [21]. This highlights that the biological effects of immunotherapy can persist for a long time, and relapse may occur following a prolonged wean.

The clinical decision to rechallenge with immunotherapy is difficult and needs to be considered on an individual basis. Invariably, the use of immunotherapy agents is currently occurring in the setting of advanced disease where the goal of prolonged cancer control is associated with improved survival. The mortality associated with the progression of cancer must be weighed up against the potentially life-threatening irAEs, and this can be a challenge for physicians with limited data available. The decision of whether to rechallenge with immunotherapy following high-grade toxicity is challenging for clinicians and patients. In general, it has been suggested that redevelopment of irAE after rechallenge is more common in patients with higher-grade irAE (grade 3 or 4). Santini *et al*. [22] recommend avoiding rechallenging patients who required hospitalization for their initial irAE, as the recurrence/new rate of irAE’s was up to 87% in these patients. Interestingly, in a study of 93 patients who were rechallenged with immunotherapy for a range of different irAEs, the recurrence of new irAE was not more severe than the original event [23].

We believe that this case series has provided further insight into the presentation and risk factors for pneumonitis in patients with NSCLC. Each of the cases of immunotherapy-induced pneumonitis illustrates the different potential patterns that may arise when immunotherapy-induced pneumonitis develops. Given the variable onset and severity of pneumonitis, we recommend that immunotherapy-induced pneumonitis must always be considered as a differential, regardless of the time of onset. Special consideration should be given to patients with underlying lung disease or a significant smoking history, given the higher risk of mortality. The decision to rechallenge patients, while difficult, is possible. Early initiation and perhaps prolonged immunosuppressive therapy may be required to optimize patient outcomes.

## Data Availability

Data sharing is not applicable to this article as no datasets were generated or analyzed during the current study.

## Abbreviations

NSCLC: Non-small-cell lung cancer
irAE: Immune-related adverse events
FDG-PET: Fluorodeoxyglucose positron emission tomography
CT: Computerized tomography
PD-1: Programmed death 1
PD-L1: Programmed death ligand 2
CTLA-4: Cytotoxic T-lymphocyte-associated protein 4
ESMO: European Society for Medical Oncology
